# Supplementary material for: Comparing linear and nonlinear finite element models of vertebral strength across the thoracolumbar spine: a benchmark from density-calibrated computed tomography
Source: Gigascience. 2025 Aug 29;14:giaf094. doi: 10.1093/gigascience/giaf094 (PMC12395960; doi:10.1093/gigascience/giaf094)
Supplement: giaf094_Supplemental_File [file giaf094_supplemental_file.docx]

**Table S1**: Relative vertebral strength compared to L1 across thoracic and lumbar levels excluding subjects with fracture (N=69, N_Fx_=91). Values represent mean percentage differences in FE-derived strength estimates relative to L1, calculated separately for males and females. Negative values indicate lower strength compared to L1. *Small sample size (n<3).

| **Vertebra** | **Overall** | **Male** | **Female** |
| --- | --- | --- | --- |
| **T1** | -80.2 | -89.5 | -64.6 |
| **T2** | -50.6 | -64.2 | -29.7 |
| **T3** | -54.2 | -68.3 | -32.6 |
| **T4** | -46.1 | -58.9 | -26.1 |
| **T5** | -38.5 | -48.6 | -22.3 |
| **T6** | -30.2 | -40.1 | -14.2 |
| **T7** | -22.7 | -31.9 | -7.6 |
| **T8** | -14.9 | -23.1 | -1.3 |
| **T9** | -9.7 | -18.5 | 4.6 |
| **T10** | -12.8 | -23.2 | 4.2 |
| **T11** | 3.7 | 1.2 | 7.6 |
| **T12** | 0.8 | 1.1 | 0.6 |
| **L2** | 6.5 | 6.3 | 6.7 |
| **L3** | 13.3 | 13.2 | 13.5 |
| **L4** | 20.0 | 16.1 | 23.7 |
| **L5** | 27.9 | 18.3 | 36.4 |
| **L6** | 21.4 | 11.8 | - |
