# Supplementary material for: Comparing linear and nonlinear finite element models of vertebral strength across the thoracolumbar spine: a benchmark from density-calibrated computed tomography
Source: Gigascience. 2025 Aug 29;14:giaf094. doi: 10.1093/gigascience/giaf094 (PMC12395960; doi:10.1093/gigascience/giaf094)
Supplement: giaf094_Authors_Response_To_Reviewer_Comments_Original_Submission [file giaf094_authors_response_to_reviewer_comments_original_submission.pdf]

Author's Response To Reviewer Comments

We thank the reviewers for their thoughtful feedback on our manuscript “Comparing Linear and Nonlinear Finite Element Models of Vertebral Strength Across the Thoracolumbar Spine: A Benchmark from Density-Calibrated Computed Tomography,” submitted to GigaScience. Below, we address each comment in turn. Reviewer comments are indicated by "Reviewer [#]", our responses follow each comment indicated by "Response", and changes made to the manuscript are indicated by "Line [##]".

Reviewer 1: The study presents a novel technique that could advance vertebral strength estimations using FE analysis. The authors clearly articulate the motivation for open benchmarking, covering spinal regions (T1-L6) that are not typically included in similar studies. The description and availability of both linear and nonlinear models support the method's broad utility. I value the authors' effort to share data and open-source resources, which enhances reproducibility. Suggestions are recommended to enhance the manuscript and clarify/expand some sections for future readers.

Response: Thank you for the valuable suggestions which we have integrated in the revised manuscript.

Reviewer 1: (Lines 122-132) The justification for choosing 0.2% axial strain as the calibration threshold is somewhat empirical and based on only three representative samples (low, medium, and high vBMD). Please, expand on how representative these three samples are of the entire cohort and whether additional samples were tested to confirm generalizability.

Response: We initially selected three representative samples to determine the 0.2% axial strain threshold, which was then applied to the full dataset. The resulting strong correlation ($R = 0.96$) supports its generalizability. We have clarified this in the manuscript and acknowledge a slight underestimation in high-strength samples, suggesting that a fixed threshold may not fully capture all cases.

Line 153-156: Importantly, the few outliers where linear analysis overestimated strength occurred only in individuals with high strength who are not considered at risk. This highlights that a fixed 0.2% threshold may not fully capture variability across all bone strengths, though it provides a practical and consistent approximation for individuals at risk.

Reviewer 1: (Line 151-152) The manuscript notes that T12 (+2.2%) and T9 (-2.1%) exhibited the smallest deviation from L1, suggesting their potential as alternative targets. In addition to calculating these deviations, was any further analysis performed to support this conclusion? Consider expanding on whether more extensive validation or simulations would be necessary to robustly support T12 and T9 as substitutes for L1.

Response: To support this observation, we added an analysis comparing vertebral strength at T9, T12, and L1 between individuals with and without vertebral fractures. Strength at all three levels was significantly lower in those with fractures (T9: $p = 0.020$, T12: $p = 0.012$, L1: $p = 0.005$), supporting their relevance as alternative targets for opportunistic assessment. These results are now included in the revised manuscript along with a summary figure. To further validate the comparability of T12 and T9 to L1, we also performed a secondary analysis restricted to individuals without vertebral fractures. Under these conditions, T12 showed only a 0.8% deviation from L1 (Supplementary Table S1), confirming its suitability as a surrogate level.

Line 187-191: When restricting the analysis to non-fracture cases, T12 showed only a 0.8% deviation from L1 (Supplementary Table S1), further confirming its suitability as a reference level. Consistent with this, vertebral strength was significantly lower at T9 ($p < 0.05$), T12 ($p = 0.01$), and L1 ($p < 0.01$) in individuals with fractures compared to individuals without fractures (Fig. 6C).

Line 429: Differences in vertebral strength between individuals with and without vertebral fractures were assessed using one-sided Mann–Whitney U tests.

Reviewer 1: (Lines 198-200) The description of cortical bone modeling is vague. It is not clear if the cortical bone was not modeled explicitly, but was implicitly accounted for. Clarification would be appreciated. Additionally, please comment on whether the method leads to under- or overestimation of strength in areas where cortical bone is predominant. Is this a limitation that might impact model predictions?

Response: Cortical and trabecular regions were segmented separately and are provided as part of the dataset. However, both were assigned the same density-based material property law. Thus, while cortical bone was included geometrically, it was not modeled with distinct material properties. This approach may slightly underestimate strength in regions dominated by cortical bone due to the material assignment. We have clarified this point and acknowledged the limitation in the revised manuscript.

Line 239-246: Both models assign material properties voxel-wise based on apparent density from calibrated CT scans, implicitly capturing cortical bone through higher density values—given CT's limited resolution and partial volume effects that often overestimate cortical thickness [24]. While we provide cortical bone segmentations based on a previous approach [25], these were not used to assign different material properties. Instead, the same density-based material law was applied across all bone tissue. This approach may lead to an underestimation of strength in regions where cortical bone predominates but aligns with current practices in voxel-based finite element modeling of CT data [19].

Reviewer 1: (Line 314) Is there a specific reason why the posterior elements were included in the segmentation process? Previous studies have often omitted these structures from their models. A brief justification for their inclusion in the present work would be helpful.

Response: Posterior elements were included to preserve anatomical completeness, as their removal would alter vertebral geometry and could introduce artificial weak points. All loading was applied exclusively to the vertebral body to simulate standard axial compression, minimizing their influence on load-bearing outcomes.

Line 373-375: Although often excluded in clinical assessments [14], posterior elements were included to preserve anatomical completeness and avoid altering vertebral geometry.

Reviewer 1: (Lines 322-323) Are there any references or prior studies that support the selection of the specific reference tissues used for phantomless calibration?

Response: We previously evaluated ten different reference tissue combinations for phantomless calibration and identified the most robust set for our purposes (Matheson et al., 2024 [4]). This citation has been added to the manuscript to support the selection.

Reviewer 1: (Lines 349-356) While equations for modulus and yield stress are provided, a short explanation of how these equations compare to other published models and why they were chosen could be more clearly included.

Response: We now clarify that we chose a density–modulus and density–strength relationship similar to Kopperdahl et al. (2002), which applies power-law fits directly to QCT-calibrated density and enables voxel-wise modeling across the vertebra without segmenting cortical and trabecular compartments. These equations have been validated in large-scale spinal FE studies. Compared to earlier relationships by Rho et al. (1995) or Morgan et al. (2003), which use ultrasonic or high-resolution mechanical testing of isolated specimens (often trabecular only), our model is optimized for use with in vivo QCT data and consistent with current clinical FEA tools.

Line 411-417: For the linear model, the elastic modulus (E) was assigned voxel-wise based on the density–modulus relationship described by Kopperdahl, Morgan [22], E = 2980 * ρ_QCT^1.05, where ρQCT is the calibrated apparent density (g/cm³). These QCT-based power-law relationships differ from earlier ultrasonic or micro-CT-derived models [45, 46] by enabling voxel-wise assignment directly from clinical-resolution density.

Reviewer 1: (Lines 361-373) The explanation of the simulation procedure, while valuable, does not clearly state whether it was performed solely on the L4 vertebra (described as the reference image) or applied individually to each vertebral body. Please clarify this point. Additionally, although the loading and boundary conditions are described, the manuscript lacks detail on how endplate irregularities or variations in vertebral alignment were addressed.

Response: We clarified that the simulation procedure was applied individually to each vertebra. To ensure consistent alignment across spinal levels, vertebral body masks were rigidly registered to a reference-aligned vertebra using ICP and principal component scaling. Additionally, we added a simulated compliant PMMA layer to the endplates to address anatomical variation and mitigate the effects of irregular surfaces during load application.

Line 427-431: All vertebral body masks were individually rigidly registered to a reference-aligned vertebra using an iterative closest point (ICP) transform. A single reference image (of the L4) was used and adjusted for vertebra of varying size and spinal level through principal component scaling ensuring consistent alignment of vertebral orientation prior to load application.

Line 439-443: A 3-voxel-thick polymethylmethacrylate (PMMA) material was added to the superior and inferior surfaces, assigned a yield stress of 70.0 MPa and an elastic modulus of 2500 MPa and Poisson's ratio of 0.3 to ensure consistent load distribution [23]. This compliant layer mitigates the effects of endplate irregularities and anatomical variability by distributing loads evenly across the interface.

Reviewer 1: (Line 387) For the failure load calculation using the stiffness-based method, which specific vertebrae were used to measure height? Please clarify whether height measurements were taken from all vertebrae in the cohort, only from those included in the force analysis, or from a subset.

Response: Vertebral height was measured individually for each segmented vertebra included in the analysis. This has been clarified in the manuscript as seen below:

Line 456: H is the vertebral height measured from the segmented mesh of each vertebra.

Reviewer 1: (Lines 397-399) The "graph model" approach for intervertebral strength normalization is not explained in detail. While it appears that this method corresponds to the analysis presented in Figure 6, this connection is not clearly stated in the text.

Response: We now added a reference to the graph model in the results section and expanded the explanation of how the model was constructed to clarify its role in the analysis shown in Figure 6.

Line 171-173: The approach first assessed within-subject differences between adjacent vertebrae using a graph model (Fig. 6A), while the second used the population-average L1 strength as a reference (Fig. 6B).

Line 466-472: First, within-subject comparisons were performed between adjacent vertebrae to calculate strength ratios. These ratios were then used to construct a graph model in which each node represents a vertebral level and edges represent the average relative strength between anatomically adjacent, non-fractured vertebrae across subjects. By using average strength ratios instead of individual subject-level paths, the model enables straightforward accumulation of relative strengths to a common reference level (e.g., L1), facilitating comparisons across the entire spine.

Reviewer 1: (Lines 122-144) In the section Linear models approximate nonlinear vertebral strength estimates, it is unclear how the nonlinear model itself was validated. The manuscript does not reference any experimental or literature-based benchmarks to support the accuracy of the nonlinear failure load predictions. Please clarify whether any validation against in vitro or in vivo vertebral failure data was performed or cited. If such validation is lacking, this should be acknowledged as a limitation and discussed in terms of its potential impact on the interpretation of the results.

Response: While we did not directly validate the nonlinear model experimentally in this study, we followed

a previously established approach by Wang et al. (2012), which demonstrated strong agreement with experimental failure data. This reference has now been added to the manuscript to support the method used.

Line 238-239: A similar nonlinear modeling approach demonstrated strong agreement with experimental data ($R^2 = 0.85$) [23].

Reviewer 1 (minor suggestions): Terminology: The term "phantomless calibration" is well-used, but a brief definition upfront (in Abstract or Background) would help readers unfamiliar with the concept.

Response: We added the concept of phantomless calibration in the background.

Line 51-53: Phantomless calibration techniques enable estimation of bone density from these scans by using internal reference tissues, eliminating the need for a physical calibration phantom during image acquisition [3, 4].

Reviewer 1 (minor suggestions): (Line 59) The word "transparent" refers to a clearer modeling workflow?

Response: We revised the sentence to improve clarity and avoid ambiguity in the use of "transparent."

Line 82: These resources cover all thoracic and lumbar vertebrae (T1–L5) and are intended to fill a critical gap in vertebral strength modeling, enabling open and reproducible comparison of FE analysis pipelines across research groups.

Reviewer 1 (minor suggestions): (Lines 87-89) Consider relocation of the statement ("By providing these outputs, we offer a ready-to-use reference..."), which seems confusing and cuts the flow of the text.

Response: We revised the sentence to improve clarity and flow within the paragraph.

Line 92: These outputs serve as a ready-to-use reference for future studies aiming to develop or validate simplified surrogate models, eliminating the need to rerun high-fidelity simulations.

Reviewer 1 (minor suggestions): FIGURES: Ensure axis labels, units, and legends in all figures (especially Fig. 4 and Fig. 6) are visible and explained.

Response: We revised the caption of Figure 6 to better describe the presented data and ensure clarity in axis labels, units, and legends.

Reviewer 1 (minor suggestions): FIGURE 3A - C. The subtitle titles could lead to misinterpretation or confusion about what is being described.

Response: We updated the captions for Figures 3 and 5 to clarify the content and avoid potential misinterpretation.

Reviewer 2: The study uses an open-source dataset collected in a population representative of those who would benefit from opportunistic screening and included physiological variation (i.e. contrast enhanced images and pre-existing fracture), alongside validation of density and FE assessment calibration methods. The methods are described in detail, including software versioning schemes, and links to the software sources as relevant for use in replicating methods. Additionally, the enhanced dataset is being included alongside the publication. The primary purpose of this study was to prepare and make available a public dataset for use in continued testing and development of opportunistic screening methods. The data appears to be conservatively analyzed as such, and the authors make notes of existing limitations of the population and sample characteristics where applicable. Additionally, the phantomless calibration technique is validated within this dataset prior to use in support of the "generalizability of the approach" (178), though the applied sample for this is relatively small (n=17 with in-scan phantoms). The manuscript is well-written and easy to understand but I have a few suggestions and comments that need to be addressed.

Response: We appreciate the positive feedback and have addressed the specific suggestions and comments below.

Reviewer 2: The data are well-controlled for the study cohort, however as mentioned by the authors (228-232), this cohort is biased towards individuals with pre-existing skeletal fragility, as indicated by the average lumbar T-score as assessed by DXA falling in the osteopenic range (-1.5, Table 1). Beyond this, the authors made use of multiple validated calibration techniques to support the use of their internal calibration scheme, as well as analysis of potential confounding variables such as contrast enhanced CT scans. Relative vertebral strength analysis (Figure 6, Table 2), however does not appear to be analyzed with respect to the fractures mentioned as present throughout the cohort (193). While differences in strength may be primarily explained by density or size, it is possible that the incidence of pre-existing fracture occurring in the thoracolumbar segment may influence adaptation of the other vertebrae in the region [1][2][3], and as such analysis for fracture inclusion may be warranted.

Response: We agree that pre-existing fractures could influence regional strength distributions. To assess this, we re-analyzed intervertebral strength ratios after excluding subjects with vertebral fractures. T12 remained the best alternative to L1, suggesting this finding is robust even when potential fracture-related adaptations are excluded. We have added this analysis as Supplementary Table S1 and updated the manuscript accordingly.

Line 187-189: When restricting the analysis to non-fracture cases, T12 showed only a 0.8% deviation from L1 (Supplementary Table S1), further confirming its suitability as a reference level.

Reviewer 2: The use of standardized FE modeling techniques supports the goal for reproducibility of assessment in clinical FE modeling. While the authors made efforts to enhance the reproducibility and generalizability of the dataset, they themselves note that the source population is not necessarily descriptive of a general population (lines 227-232). Though this population is representative of those indicated for opportunistic screening, the development of risk curves necessitates the inclusion of healthy individuals, and follow-up analysis to fully flesh out the use of opportunistic FE in clinical settings, however this analysis would require a much larger cohort, and are outside the scope of the current manuscript.

Response: We agree. The current dataset is intended as a benchmark for method development and comparison. A larger, more representative cohort is currently being collected to support future analyses such as population-based risk curve development. We clarified this point in the manuscript.

Line 294-296: In addition, while the dataset is appropriate for screening applications, its lack of representation of healthy individuals limits the development of generalized risk thresholds.

Reviewer 2: Further, while 'voxel-models' are typically regarded as standard, tetrahedral element models may generally provide better representation of complex biological geometries [4]. All approaches to FE have drawbacks, and tetrahedral models may be less-optimal solutions compared to hexahedral elements for convergence and the possibility of artificial stiffening, the high prevalence of osteophytes and degradation [5], particularly in older populations where screening is indicated, may warrant the use of tetrahedral elements which capture the intricacies of vertebral geometry that impact FE derived strength [6]. While again potentially outside the scope of this study, it might be noted as an additional formulative variable for FE approaches to estimating fracture risk.

Response: We agree that all meshing approaches involve trade-offs. Voxel-based hexahedral elements are widely used due to their reproducibility and ease of automation but may limit geometric fidelity in anatomically complex regions. We have noted this in the manuscript and acknowledged it as a consideration for future methodological developments.

Line 290: We also used voxel-based 8-node linear hexahedral elements, which offer high reproducibility but may limit geometric fidelity in regions with complex morphology, such as vertebral margins affected by osteophyte formation, compared to tetrahedral meshes or higher order elements [31].

Reviewer 2: Line 269 -> "… applications such as radiomics-driven [approach?] for opportunistic …"

Response: Thank you for catching this. We have added the missing word "approach".

Reviewer 2: As fracture prevalence is included in the dataset, it may be worthwhile to include analysis of fracture-adjacent vertebra in the selection of surrogate vertebra for L1 in opportunistic screening. Does pre-existing fracture influence which vertebrae selected, and should this decision be made on a person-to-person basis, taking into consideration the particular condition of the vertebrae available in the scan?

Response: We re-analyzed intervertebral strength ratios excluding subjects with vertebral fractures (created a new Supplementary Table S1) and also compared vertebral strength at T9, T12, and L1 between individuals with and without fractures. As expected, strength was significantly lower at all three levels in those with fractures (T9: $p = 0.020$, T12: $p = 0.012$, L1: $p = 0.005$), supporting their use as alternative sites. These findings are included in the revised manuscript (and below for convenience) along with a summary figure (Fig. 6C).

Line 187-191: When restricting the analysis to non-fracture cases, T12 showed only a 0.8% deviation from L1 (Supplementary Table S1), further confirming its suitability as a reference level. Consistent with this, vertebral strength was significantly lower at T9 ($p < 0.05$), T12 ($p = 0.01$), and L1 ($p < 0.01$) in individuals with fractures compared to individuals without fractures (Fig. 6C).

Table S1: Relative vertebral strength compared to L1 across thoracic and lumbar levels excluding subjects with fracture (N=69, NFx=91). Values represent mean percentage differences in FE-derived strength estimates relative to L1, calculated separately for males and females. Negative values indicate lower strength compared to L1. *Small sample size (n<3).

Vertebra	Overall	Male	Female
T1	-80.2	-89.5	-64.6
T2	-50.6	-64.2	-29.7
T3	-54.2	-68.3	-32.6
T4	-46.1	-58.9	-26.1
T5	-38.5	-48.6	-22.3
T6	-30.2	-40.1	-14.2
T7	-22.7	-31.9	-7.6
T8	-14.9	-23.1	-1.3
T9	-9.7	-18.5	4.6
T10	-12.8	-23.2	4.2
T11	3.7	1.2	7.6
T12	0.8	1.1	0.6
L2	6.5	6.3	6.7
L3	13.3	13.2	13.5
L4	20.0	16.1	23.7
L5	27.9	18.3	36.4
L6*	21.4	11.8	-

Cookie Preference Center

We use cookies which are necessary to make our site work. We may also use additional cookies to analyze, improve and personalize our content and your digital experience. For more information, see our Cookie Policy.

You may choose not to allow some types of cookies. However, blocking some types may impact your experience of our site and the services we are able to offer. See the different category headings below to find out more or change your settings.

Allow all

Manage Consent Preferences

Strictly Necessary Cookies

Always active

These cookies are necessary for the website to function and cannot be switched off in our systems. They are usually only set in response to actions made by you which amount to a request for services, such as setting your privacy preferences, logging in or filling in forms. You can set your browser to block or alert you about these cookies, but some parts of the site will not then work.

Functional Cookies

These cookies enable the website to provide enhanced functionality and personalisation. They may be set by us or by third party providers whose services we have added to our pages. If you do not allow these cookies then some or all of these services may not function properly.

Performance Cookies

These cookies allow us to count visits and traffic sources so we can measure and improve the performance of our site. They help us to know which pages are the most and least popular and see how visitors move around the site.

Targeting Cookies

These cookies may be set through our site by our advertising partners. They may be used by those companies to build a profile of your interests and show you relevant adverts on other sites. If you do not allow these cookies, you will experience less targeted advertising.

Cookie List

Clear

Apply Cancel

Consent Leg.Interest

Confirm my choices

Powered by onetrust
