# Supplementary material for: Comparing linear and nonlinear finite element models of vertebral strength across the thoracolumbar spine: a benchmark from density-calibrated computed tomography
Source: Gigascience. 2025 Aug 29;14:giaf094. doi: 10.1093/gigascience/giaf094 (PMC12395960; doi:10.1093/gigascience/giaf094)
Supplement: giaf094_Reviewer_1_Report_Original_Submission — Maria Prado, Ph.D. -- 5/23/2025 [file giaf094_reviewer_1_report_original_submission.pdf]

Reviewer Report

Title: Comparing Linear and Nonlinear Finite Element Models of Vertebral Strength Across the Thoracolumbar Spine: A Benchmark from Density-Calibrated Computed Tomography

Version: Original Submission **Date:** 5/23/2025

Reviewer name: Maria Prado, Ph.D.

Reviewer Comments to Author:

The study presents a novel technique that could advance vertebral strength estimations using FE analysis. The authors clearly articulate the motivation for open benchmarking, covering spinal regions (T1-L6) that are not typically included in similar studies. The description and availability of both linear and nonlinear models support the method's broad utility. I value the authors' effort to share data and open-source resources, which enhances reproducibility.

Suggestions are recommended to enhance the manuscript and clarify/expand some sections for future readers.

(Lines 122-132) The justification for choosing 0.2% axial strain as the calibration threshold is somewhat empirical and based on only three representative samples (low, medium, and high vBMD). Please, expand on how representative these three samples are of the entire cohort and whether additional samples were tested to confirm generalizability.

(Line 151-152) The manuscript notes that T12 (+2.2%) and T9 (-2.1%) exhibited the smallest deviation from L1, suggesting their potential as alternative targets. In addition to calculating these deviations, was any further analysis performed to support this conclusion? Consider expanding on whether more extensive validation or simulations would be necessary to robustly support T12 and T9 as substitutes for L1.

(Lines 198-200) The description of cortical bone modeling is vague. It is not clear if the cortical bone was not modeled explicitly, but was implicitly accounted for. Clarification would be appreciated. Additionally, please comment on whether the method leads to under- or overestimation of strength in areas where cortical bone is predominant. Is this a limitation that might impact model predictions?

(Line 314) Is there a specific reason why the posterior elements were included in the segmentation process? Previous studies have often omitted these structures from their models. A brief justification for their inclusion in the present work would be helpful.

(Lines 322-323) Are there any references or prior studies that support the selection of the specific reference tissues used for phantomless calibration?

(Lines 349-356) While equations for modulus and yield stress are provided, a short explanation of how these equations compare to other published models and why they were chosen could be more clearly included.

(Lines 361-373) The explanation of the simulation procedure, while valuable, does not clearly state whether it was performed solely on the L4 vertebra (described as the reference image) or applied individually to each vertebral body. Please clarify this point. Additionally, although the loading and boundary conditions are described, the manuscript lacks detail on how endplate irregularities or

variations in vertebral alignment were addressed.
(Line 387) For the failure load calculation using the stiffness-based method, which specific vertebrae were used to measure height? Please clarify whether height measurements were taken from all vertebrae in the cohort, only from those included in the force analysis, or from a subset.
(Lines 397-399) The "graph model" approach for intervertebral strength normalization is not explained in detail. While it appears that this method corresponds to the analysis presented in Figure 6, this connection is not clearly stated in the text.
(Lines 122-144) In the section Linear models approximate nonlinear vertebral strength estimates, it is unclear how the nonlinear model itself was validated. The manuscript does not reference any experimental or literature-based benchmarks to support the accuracy of the nonlinear failure load predictions. Please clarify whether any validation against in vitro or in vivo vertebral failure data was performed or cited. If such validation is lacking, this should be acknowledged as a limitation and discussed in terms of its potential impact on the interpretation of the results.
Minor suggestions:
Terminology: The term "phantomless calibration" is well-used, but a brief definition upfront (in Abstract or Background) would help readers unfamiliar with the concept.
(Line 59) The word "transparent" refers to a clearer modeling workflow?
(Lines 87-89) Consider relocation of the statement ("By providing these outputs, we offer a ready-to-use reference..."), which seems confusing and cuts the flow of the text.
FIGURES: Ensure axis labels, units, and legends in all figures (especially Fig. 4 and Fig. 6) are visible and explained.
FIGURE 3A - C. The subtitle titles could lead to misinterpretation or confusion about what is being described.

Methods

Are the methods appropriate to the aims of the study, are they well described, and are necessary controls included? Choose an item.

Conclusions

Are the conclusions adequately supported by the data shown? Choose an item.

Reporting Standards

Does the manuscript adhere to the journal’s guidelines on [minimum standards of reporting?](#) Choose an item.

Choose an item.

Statistics

Are you able to assess all statistics in the manuscript, including the appropriateness of statistical tests used? Choose an item.

Quality of Written English

Please indicate the quality of language in the manuscript: Choose an item.

Declaration of Competing Interests

Please complete a declaration of competing interests, considering the following questions:

- Have you in the past five years received reimbursements, fees, funding, or salary from an organisation that may in any way gain or lose financially from the publication of this manuscript, either now or in the future?
- Do you hold any stocks or shares in an organisation that may in any way gain or lose financially from the publication of this manuscript, either now or in the future?
- Do you hold or are you currently applying for any patents relating to the content of the manuscript?
- Have you received reimbursements, fees, funding, or salary from an organization that holds or has applied for patents relating to the content of the manuscript?
- Do you have any other financial competing interests?
- Do you have any non-financial competing interests in relation to this paper?

If you can answer no to all of the above, write 'I declare that I have no competing interests' below. If your reply is yes to any, please give details below.

I declare that I have no competing interests

I agree to the open peer review policy of the journal. I understand that my name will be included on my report to the authors and, if the manuscript is accepted for publication, my named report including any attachments I upload will be posted on the website along with the authors' responses. I agree for my report to be made available under an Open Access Creative Commons CC-BY license (http://creativecommons.org/licenses/by/4.0/). I understand that any comments which I do not wish to be included in my named report can be included as confidential comments to the editors, which will not be published.

Choose an item.

To further support our reviewers, we have joined with Publons, where you can gain additional credit to further highlight your hard work (see: https://publons.com/journal/530/gigascience). On publication of this paper, your review will be automatically added to Publons, you can then choose whether or not to claim your Publons credit. I understand this statement.

Yes Choose an item.
