# Supplementary material for: Comparing linear and nonlinear finite element models of vertebral strength across the thoracolumbar spine: a benchmark from density-calibrated computed tomography
Source: Gigascience. 2025 Aug 29;14:giaf094. doi: 10.1093/gigascience/giaf094 (PMC12395960; doi:10.1093/gigascience/giaf094)
Supplement: giaf094_Reviewer_2_Report_Original_Submission — Karan Devane -- 5/27/2025 [file giaf094_reviewer_2_report_original_submission.pdf]

Reviewer Report

Title: Comparing Linear and Nonlinear Finite Element Models of Vertebral Strength Across the Thoracolumbar Spine: A Benchmark from Density-Calibrated Computed Tomography

Version: Original Submission **Date:** 5/27/2025

Reviewer name: Karan Devane

Reviewer Comments to Author:

The study uses an open-source dataset collected in a population representative of those who would benefit from opportunistic screening and included physiological variation (i.e. contrast enhanced images and pre-existing fracture), alongside validation of density and FE assessment calibration methods. The methods are described in detail, including software versioning schemes, and links to the software sources as relevant for use in replicating methods. Additionally, the enhanced dataset is being included alongside the publication. The primary purpose of this study was to prepare and make available a public dataset for use in continued testing and development of opportunistic screening methods. The data appears to be conservatively analyzed as such, and the authors make notes of existing limitations of the population and sample characteristics where applicable. Additionally, the phantomless calibration technique is validated within this dataset prior to use in support of the "generalizability of the approach" (178), though the applied sample for this is relatively small (n=17 with in-scan phantoms). The manuscript is well-written and easy to understand but I have a few suggestions and comments that need to be addressed.

The data are well-controlled for the study cohort, however as mentioned by the authors (228-232), this cohort is biased towards individuals with pre-existing skeletal fragility, as indicated by the average lumbar T-score as assessed by DXA falling in the osteopenic range (-1.5, Table 1). Beyond this, the authors made use of multiple validated calibration techniques to support the use of their internal calibration scheme, as well as analysis of potential confounding variables such as contrast enhanced CT scans. Relative vertebral strength analysis (Figure 6, Table 2), however does not appear to be analyzed with respect to the fractures mentioned as present throughout the cohort (193). While differences in strength may be primarily explained by density or size, it is possible that the incidence of pre-existing fracture occurring in the thoracolumbar segment may influence adaptation of the other vertebrae in the region [1][2][3], and as such analysis for fracture inclusion may be warranted.

The use of standardized FE modeling techniques supports the goal for reproducibility of assessment in clinical FE modeling. While the authors made efforts to enhance the reproducibility and generalizability of the dataset, they themselves note that the source population is not necessarily descriptive of a general population (lines 227-232). Though this population is representative of those indicated for opportunistic screening, the development of risk curves necessitates the inclusion of healthy individuals, and follow-up analysis to fully flesh out the use of opportunistic FE in clinical settings, however this analysis would require a much larger cohort, and are outside the scope of the current manuscript. Further, while 'voxel-models' are typically regarded as standard, tetrahedral element models may generally provide better representation of complex biological geometries [4]. All approaches to FE have

drawbacks, and tetrahedral models may be less-optimal solutions compared to hexahedral elements for convergence and the possibility of artificial stiffening, the high prevalence of osteophytes and degradation [5], particularly in older populations where screening is indicated, may warrant the use of tetrahedral elements which capture the intricacies of vertebral geometry that impact FE derived strength [6]. While again potentially outside the scope of this study, it might be noted as an additional formulative variable for FE approaches to estimating fracture risk.
Line 269 -> "… applications such as radiomics-driven [approach?] for opportunistic …" As fracture prevalence is included in the dataset, it may be worthwhile to include analysis of fracture-adjacent vertebra in the selection of surrogate vertebra for L1 in opportunistic screening. Does pre-existing fracture influence which vertebrae selected, and should this decision be made on a person-to-person basis, taking into consideration the particular condition of the vertebrae available in the scan?
[1] https://pmc.ncbi.nlm.nih.gov/articles/PMC8752702/
[2] https://academic.oup.com/jbmr/article/39/12/1744/7825427
[3] https://pmc.ncbi.nlm.nih.gov/articles/PMC7697376/
[4] https://www.sciencedirect.com/science/article/pii/S0021929005003568
[5] https://link.springer.com/article/10.1007/s12565-010-0080-8
[6] https://www.sciencedirect.com/science/article/pii/S1529943018306466

Methods

Are the methods appropriate to the aims of the study, are they well described, and are necessary controls included? Choose an item.

Conclusions

Are the conclusions adequately supported by the data shown? Choose an item.

Reporting Standards

Does the manuscript adhere to the journal's guidelines on minimum standards of reporting? Choose an item.

Choose an item.

Statistics

Are you able to assess all statistics in the manuscript, including the appropriateness of statistical tests used? Choose an item.

Quality of Written English

Please indicate the quality of language in the manuscript: Choose an item.

Declaration of Competing Interests

Please complete a declaration of competing interests, considering the following questions:

- Have you in the past five years received reimbursements, fees, funding, or salary from an organisation that may in any way gain or lose financially from the publication of this manuscript, either now or in the future?
- Do you hold any stocks or shares in an organisation that may in any way gain or lose financially from the publication of this manuscript, either now or in the future?
- Do you hold or are you currently applying for any patents relating to the content of the manuscript?
- Have you received reimbursements, fees, funding, or salary from an organization that holds or has applied for patents relating to the content of the manuscript?
- Do you have any other financial competing interests?
- Do you have any non-financial competing interests in relation to this paper?

If you can answer no to all of the above, write 'I declare that I have no competing interests' below. If your reply is yes to any, please give details below.

I declare that I have no competing interests.

I agree to the open peer review policy of the journal. I understand that my name will be included on my report to the authors and, if the manuscript is accepted for publication, my named report including any attachments I upload will be posted on the website along with the authors' responses. I agree for my report to be made available under an Open Access Creative Commons CC-BY license (http://creativecommons.org/licenses/by/4.0/). I understand that any comments which I do not wish to be included in my named report can be included as confidential comments to the editors, which will not be published.

Choose an item.

To further support our reviewers, we have joined with Publons, where you can gain additional credit to further highlight your hard work (see: https://publons.com/journal/530/gigascience). On publication of this paper, your review will be automatically added to Publons, you can then choose whether or not to claim your Publons credit. I understand this statement.

Yes Choose an item.
